# Maternal Telomere Length and Its Influence on Neonatal Parameters: A Potential Tool for Prenatal Screening

**DOI:** 10.3390/medicina61101755

**Published:** 2025-09-26

**Authors:** Razvan Nitu, Tiberiu Dragomir, Simona-Alina Abu-Awwad, Flavius Olaru, Carmen-Ioana Marta, Ahmed Abu-Awwad, Bogdan Sorop, Mircea Diaconu

**Affiliations:** 1 Department of Obstetrics and Gynecology, “Victor Babes” University of Medicine and Pharmacy, 300041 Timisoara, Romania; nitu.dumitru@umft.ro (R.N.); alina.abuawwad@umft.ro (S.-A.A.-A.); carmen.marta@umft.ro (C.-I.M.); diaconu.mircea@umft.ro (M.D.); 2 Clinic of Obstetrics and Gynecology, “Pius Brinzeu” County Clinical Emergency Hospital, 300723 Timisoara, Romania; 3 Medical Semiology II Discipline, Internal Medicine Department, “Victor Babes” University of Medicine and Pharmacy, Eftimie Murgu Square No. 2, 300041 Timisoara, Romania; dragomir.tiberiu@umft.ro; 4 Department XV—Discipline of Orthopedics—Traumatology, “Victor Babes” University of Medicine and Pharmacy, 300041 Timisoara, Romania; ahm.abuawwad@umft.ro; 5 Research Center University Professor Doctor Teodor Șora, “Victor Babes” University of Medicine and Pharmacy, 300041 Timisoara, Romania

**Keywords:** maternal telomere length, neonatal outcomes, Apgar score, birth weight, gestational age, fetal development, prenatal screening, biological aging, telomere dynamics, perinatal biomarkers

## Abstract

*Background and Objectives*: Maternal telomere length (TL) has been proposed as a potential biomarker of biological aging and pregnancy outcomes, yet evidence in Central and Eastern European populations remains scarce. This study aimed to investigate the association between maternal TL and neonatal parameters in a clinically healthy cohort. *Materials and Methods*: We conducted a prospective observational study including 134 mother–infant pairs at the “Pius Brînzeu” Emergency County Clinical Hospital, Timișoara. All deliveries were performed by cesarean section for maternal indications unrelated to fetal condition. Maternal blood samples were collected at admission, and relative TL was measured by quantitative PCR. Neonatal outcomes included birth weight, length, head circumference, gestational age, and Apgar scores. *Results*: Longer maternal TL was positively correlated with birth weight (r = 0.515, *p* < 0.001), length (r = 0.559, *p* < 0.001), head circumference (r = 0.468, *p* < 0.001), gestational age (r = 0.444, *p* < 0.001), and Apgar scores at 1 (r = 0.714, *p* < 0.001) and 5 min (r = 0.684, *p* < 0.001). Logistic regression showed that shorter maternal TL independently predicted suboptimal 1 min Apgar (<8), with an adjusted odds ratio of 0.68 (95% CI: 0.51–0.91). *Conclusions*: Maternal TL is strongly associated with neonatal growth and vitality measures, supporting its potential as a simple, non-invasive biomarker for perinatal risk assessment.

## 1. Introduction

In recent years, telomere biology has emerged as a fascinating frontier in understanding human development, aging, and disease susceptibility [1]. Telomeres are defined as repetitive nucleotide sequences at the ends of chromosomes that play a critical role in maintaining genomic stability [2]. Their gradual shortening with each cell division has been widely recognized as a hallmark of cellular aging [3]. While telomere length (TL) has been extensively studied in adult populations, including its association with chronic disease [4] and lifespan [5], its significance during pregnancy and fetal development remains an area of growing interest. The intrauterine environment is now known to have long-lasting effects on an individual’s health trajectory, and telomere dynamics may be a key biological marker that reflects this early life programming [6].

Maternal TL may offer important insights into fetal development. During pregnancy, a mother’s physiological and psychological states (ranging from oxidative stress to systemic inflammation) can influence not only her own telomere dynamics but potentially those of her unborn child [7]. Emerging evidence suggests that shorter maternal TL may be associated with adverse pregnancy outcomes, including intrauterine growth restriction (IUGR), low birth weight, and preterm birth [8]. Conversely, longer telomeres in mothers have been correlated with more favorable neonatal outcomes [9].

Perinatal complications represent a major cause of neonatal morbidity and mortality and most frequently include intrauterine growth restriction (IUGR), low birth weight, preterm birth, and impaired neonatal adaptation, reflected by suboptimal Apgar scores. These conditions can have immediate consequences on neonatal vitality and may influence long-term development, being closely related to maternal, placental, and environmental factors. However, findings across studies remain inconsistent, and the clinical relevance of maternal TL as a predictive marker is still under debate.

Risk factors for perinatal complications are diverse and include advanced maternal age, high or low pre-pregnancy body mass index, smoking, maternal chronic conditions such as hypertension, diabetes, and autoimmune diseases, nutritional deficiencies, infections, exposure to environmental pollutants, low socioeconomic status, and elevated maternal stress levels. These factors not only compromise maternal health but also influence the intrauterine environment, thereby increasing the risk of intrauterine growth restriction, preterm birth, and impaired neonatal adaptation.

Understanding how maternal TL may influence neonatal parameters such as birth weight, Apgar score, gestational age, and anthropometric indices could provide a non-invasive, molecular-level window into fetal well-being [10]. This has implications not only for early identification of at-risk pregnancies but also for informing public health strategies aimed at improving perinatal outcomes. In a time when personalized medicine and predictive diagnostics are reshaping obstetric care, exploring the potential of maternal TL as a biomarker is both timely and necessary.

What also makes this line of research compelling is its intersection with lifestyle, environmental, and psychosocial factors. Maternal TL is known to be influenced by age, nutrition, stress levels, socioeconomic status, and exposure to pollutants, factors that also independently impact fetal health [11]. This overlapping network of influences positions TL as a possible integrative marker, one that captures the cumulative biological toll of environmental exposures and life history and transmits it across generations [12].

Despite this promising background, large-scale human studies linking maternal TL with newborn parameters remain limited, particularly in Central and Eastern European populations. Most available data are derived from high-income countries with different sociodemographic profiles and healthcare systems. Therefore, additional research is needed to validate the utility of maternal TL in diverse clinical and population settings.

This study aims to contribute to this growing body of knowledge by investigating the association between maternal TL and key neonatal parameters. By exploring this relationship in a real-world clinical setting, we aim to gain a deeper understanding of whether maternal TL can serve as a reliable and accessible tool in prenatal risk assessment. Our findings may help to clarify the biological underpinnings of early life development and support the integration of molecular markers in routine obstetric care.

## 2. Materials and Methods

### 2.1. Study Design

This study was designed as a prospective observational study aiming to investigate the potential associations between maternal TL and neonatal parameters. The research was conducted at the “Pius Brînzeu” Emergency County Clinical Hospital in Timișoara, within the Departments of Obstetrics and Neonatology. The study was carried out over a defined period, during which participants were consecutively enrolled before delivery. Ethical approval was obtained from the institutional ethics committee (265/22 September 2021), and all participants provided written informed consent prior to inclusion.

The observational design allowed us to evaluate natural variations in TL among mothers at term and assess whether these variations correlated with specific neonatal outcomes, without introducing any intervention or deviation from standard care. Our approach focused on capturing real-life clinical data from a diverse maternity population, reflecting a broad range of maternal and neonatal health profiles.

### 2.2. Participants

A total of 134 mother–newborn pairs were included in the study. Eligibility criteria were designed to include pregnant women aged between 18 and 40 years with singleton pregnancies admitted for delivery at term (≥37 weeks of gestation) [13], who had no major fetal anomalies detected on ultrasound and no associated maternal pathologies. Eligible participants were those without obesity or chronic conditions such as cancer, autoimmune diseases, or endocrine/metabolic disorders, and who had received regular prenatal care throughout pregnancy. Only women with a history of no more than two miscarriages, negative COVID-19 status in the past year, and no prior psychiatric, thromboembolic, or infectious diseases (HBV, HCV, HIV/AIDS) were included.

Exclusion criteria included: history of drug or alcohol abuse, psychiatric disorders, thromboembolic events, participation in another clinical study within the past 3 months, adverse reactions to phlebotomy, ongoing infections, or pregnancies resulting from assisted reproductive techniques. Also, women who smoke were excluded. Also, patients were excluded from the analysis if they showed CRP ≥ 3 mg/L, IL-6 ≥ 7 pg/mL, WBC < 4.5 or >11 × 10^9^/L, fasting glucose ≥ 100 mg/dL or HbA1c ≥ 5.7%, ferritin < 15 or >300 ng/mL, vitamin D < 20 ng/mL, or TSH outside the 0.4–4.0 µIU/mL range, to prevent marked inflammatory, metabolic, or endocrine disturbances from skewing the relationship between the inflammatory milieu and TL.

Participants were enrolled upon admission to the maternity ward, where delivery was carried out exclusively by elective cesarean section for maternal indications unrelated to fetal condition. This decision ensured a uniform mode of delivery and minimized variability related to intrapartum factors such as labor stress or complications, thereby reducing heterogeneity in neonatal outcomes. We acknowledge, however, that this restriction may limit generalizability and could introduce a degree of selection bias. Following informed consent, maternal blood samples were collected for TL analysis. Relevant clinical and demographic information was extracted from medical records and supplemented by direct interviews when needed.

### 2.3. Variables and Data Collection

Upon admission for delivery each participant provided a 5 mL peripheral-venous sample into an EDTA tube. Within two hours the sample was centrifuged and the buffy coat isolated; genomic DNA was extracted with a silica-membrane kit (QIAamp DNA Blood Mini, Qiagen, Hilden, Germany) and quantified spectrophotometrically using NanoDrop 2000 (Thermo Fisher Scientific, Waltham, MA, USA). Relative TL was determined in triplicate by monochrome quantitative PCR on an ABI 7500 platform (Applied Biosystems, Foster City, CA, USA), following Cawthon’s T/S method, with 36B4 as the single-copy reference gene. A pooled genomic DNA sample from healthy adult donors was included on each plate as a calibrator reference, against which all samples were normalized. Standard curves were generated from a 5-point serial dilution (1:5) of the calibrator DNA, and reaction efficiency was maintained between 95% and 103%, consistent with established qPCR quality standards. Inter-plate coefficients of variation were kept below 7%. The mean of the triplicates was carried forward as the individual TL value. Intra-assay replicates were performed in triplicate for each sample, with a coefficient of variation maintained below 5%.

Demographic and obstetric data (including age, body mass index (BMI), parity, place of residence, level of education, and employment status) were extracted from the electronic medical records and confirmed through a brief bedside interview. At the same time as the telomere sampling, venous blood was collected to assess several routine laboratory parameters relevant to telomere biology.

High-sensitivity C-reactive protein (CRP) and serum glucose levels were measured using immunoturbidimetric and hexokinase methods, respectively with the architect ci4100 analyzer (Abbott Diagnostics, Abbott Park, IL, USA). Interleukin-6 (IL-6) concentrations were determined using a sandwich ELISA (R&D Systems Minneapolis, MN, USA), while a complete blood count with 5-part differential was performed with the Sysmex XN-1000 analyzer (Sysmex Corporation, Kobe, Japan). Glycated hemoglobin (HbA1c) was measured via high-performance liquid chromatography (HPLC) using the Bio-Rad D-100 system (Bio-Rad Laboratories, Hercules, CA, USA). Ferritin levels were assessed through a chemiluminescent microparticle immunoassay (Abbott Diagnostics, Abbott Park, IL, USA), and 25-hydroxyvitamin D concentrations were quantified via electrochemiluminescence (Roche Diagnostics, Mannheim, Germany). Thyroid-stimulating hormone (TSH) was measured using a third-generation immunoassay (Roche Diagnostics, Mannheim, Germany).

Immediately after birth the attending neonatologist documented: birthweight, crown–heel length, head circumference, Apgar scores at one and five minutes, and gestational age. Data entry occurred within one hour of delivery on a standardized electronic form, and a second clinician verified all fields.

By harmonizing the collection time-point (admission for term delivery), using calibrated instruments and applying strict exclusion thresholds, we ensured that both exposure (maternal TL) and outcomes (neonatal metrics) were measured under tightly controlled, physiologically comparable conditions.

To minimize bias, laboratory personnel performing the telomere length assays were blinded to maternal clinical characteristics and neonatal outcomes. Likewise, neonatologists documenting Apgar scores and anthropometric parameters were blinded to maternal TL values. This ensured that outcome assessments and laboratory measurements were conducted independently.

### 2.4. Statistical Analysis

All analyses were conducted using GraphPad Prism 9 and MedCalc 22.0. GraphPad Prism was used for descriptive and correlation analyses, while MedCalc was employed for multivariable modeling. We first performed descriptive analyses, reporting means, standard deviations, and two-sided 95% confidence intervals to characterize the distribution of the data. Normality of distributions was assessed using the Shapiro–Wilk test in Prism, guiding the choice between parametric and non-parametric analyses. For variables following a normal distribution, Pearson correlation coefficients were calculated; when normality assumptions were not met, Spearman’s rank correlation was applied.

The heart of the inferential work was a multiple linear regression built in MedCalc, with maternal TL as the independent predictor variable and the full suite of neonatal measures (birth weight, length, head circumference, Apgar scores at one and five minutes, and gestational age) as dependent outcomes, all adjusted for maternal age, BMI, and parity. Model assumptions were systematically evaluated: homoscedasticity was examined through residual plots, independence of errors was assessed with the Durbin–Watson statistic, and variance-inflation factors were used to exclude collinearity. Statistical significance was set at *p* < 0.05 (two-tailed), and where multiple comparisons might stack up, the false-discovery rate was controlled with the Benjamini–Hochberg procedure. To validate the robustness of the findings, a post hoc power analysis was performed for the strongest correlation (maternal TL versus 1 min Apgar score), yielding a power of 0.92, which supports the reliability of this association.

In addition, partial correlation analyses adjusting for maternal age, BMI, and parity were conducted to confirm the robustness of associations. To further explore potential non-linear relationships, maternal TL was also examined as a continuous predictor using restricted cubic spline regression. Tertile-based stratification was applied as a hypothesis-driven approach to visualize dose–response trends and investigate potential threshold effects.

## 3. Results

Baseline demographic characteristics of the maternal cohort are summarized in Table 1. The mean maternal age was 28.6 ± 2.2 years, and the average body mass index (BMI) was 23.1 ± 2.0 kg/m^2^, reflecting a predominantly normal-weight population. The median parity was 1.0, with an interquartile range (IQR) of 0.0 to 1.0, indicating that most participants were either primiparous or had a single previous birth.

In terms of educational attainment, most women had completed higher education, with 56.0% holding a university degree and 17.9% having postgraduate qualifications. A smaller proportion, 26.1%, reported having only completed high school. The population was predominantly urban, with 79.9% residing in urban areas, while 20.1% lived in rural communities. Regarding employment status, 71.6% were employed, 17.2% were unemployed, and 11.2% were students at the time of data collection. These demographic data reflect a relatively young, educated, and socioeconomically active maternal population.

Table 2 summarizes the distribution of biological markers in the cohort, including inflammatory, hematologic, metabolic, endocrine, micronutrient, iron-storage, and telomere-related parameters. For each marker, mean values, standard deviations, and 95% confidence intervals are reported to indicate both central tendency and variability within the cohort.

The descriptive statistics indicated low levels of systemic inflammation, normal glycemic control, and thyroid function within the reference range. Ferritin and vitamin D showed the greatest variability, suggesting inter-individual differences in iron stores and vitamin D status. Maternal TL, expressed as relative T/S ratio, also showed marked variability (mean = 0.72 ± 0.60, range 0.47–1.93), consistent with heterogeneity in biological aging among participants. These descriptive data provided the basis for subsequent multivariate analyses assessing the relationship between maternal TL and neonatal outcomes.

Descriptive statistics for the neonatal cohort are presented in Table 3. The mean birth weight was 3284 ± 166 g, while the average birth length was 52.4 ± 1.7 cm. Head circumference had a mean value of 34.4 ± 1.2 cm, consistent with normative data for term infants. Neonatal adaptation was generally favorable, as reflected by an average Apgar score of 8.2 ± 0.8 at 1 min and 9.2 ± 0.7 at 5 min. The gestational age at birth ranged between 37 and 41 weeks, with a mean of 38.9 ± 0.9 weeks, confirming that all births occurred at term. These values indicate a clinically stable and homogeneous neonatal population, suitable for evaluating associations with maternal biological parameters.

To test whether TL acts as a linear predictor or shows meaningful cut-offs, the cohort was split into three equally sized bands (short, mid-range, and long telomeres) while preserving the overall mean and spread- Table 4. A clear dose–response pattern emerged: moving from the shortest to the longest band, Apgar scores at one and five minutes improved steadily, and newborns were incrementally heavier, slightly longer, and had marginally larger head circumferences. Gestational age inched upward as well, though without reaching statistical significance, suggesting that TL may influence the quality of neonatal adaptation more than the duration of pregnancy. Classic one-way ANOVA was applied where normality held, with Kruskal–Wallis used for non-Gaussian variables; multiple testing was controlled via the Benjamini–Hochberg procedure. The most pronounced effect was observed for the 1 min Apgar score, with a threshold emerging around a maternal T/S ratio of 0.85. Beyond this point, further increases in telomere length were associated with only minimal additional improvements in neonatal outcomes.

Significant positive correlations were identified between maternal TL and all examined neonatal parameters, as presented in Table 5. The strongest association was observed with the Apgar score at 1 min (r = 0.714, *p* < 0.0001), suggesting a potential link between maternal cellular aging and immediate neonatal adaptation. Moderate correlations were also found with birth length (r = 0.559), Apgar score at 5 min (r = 0.648), and birth weight (r = 0.515), all statistically significant (*p* < 0.0001). Head circumference demonstrated a weaker, yet still meaningful correlation (r = 0.468, *p* < 0.0001). These results support the hypothesis that longer maternal telomeres may be associated with improved fetal growth and early neonatal outcomes. While causality cannot be established, the consistency and strength of the associations highlight the potential of maternal TL as a biomarker of perinatal health.

To assess the robustness of our findings, we conducted a sensitivity analysis excluding extreme values (upper and lower 5%) of maternal TL and neonatal outcomes. The direction and magnitude of the correlations remained unchanged, supporting the stability of the associations.

To align the statistics with everyday clinical decision-making, the 1 min Apgar score was recoded into a binary outcome: sub-optimal adaptation (<8) versus good adaptation (≥8). Within the 134 mother–infant pairs, 27 newborns (≈20%) fell into the sub-optimal group- Table 6.

A binary logistic model showed that each 0.25-unit rise in the maternal T/S ratio cut the odds of a low Apgar by roughly one-third. This protective effect persisted—even after controlling for maternal age, BMI and parity—underscoring that short maternal telomeres are an independent risk marker for early neonatal compromise.

The adjusted model achieved an area under the ROC curve of 0.78, and a Hosmer–Lemeshow test (*p* = 0.62) confirmed good calibration, suggesting that maternal TL could serve as a clinically meaningful, easy-to-measure addition to perinatal risk stratification.

## 4. Discussion

A total of 134 mother–newborn pairs were included, providing a demographically uniform and well-characterized sample for examining how maternal telomere length relates to neonatal outcomes. The mean maternal age was 28.6 ± 2.2 years, and we intentionally kept age variability narrow given the sensitivity of telomere length to aging. Women with pre-pregnancy obesity were excluded because of its known association with accelerated telomere shortening [14]. To further minimize confounding, we also excluded participants with other established drivers of telomere attrition such as active smoking [15], chronic infections [16], or metabolic disease [17], resulting in a cohort that better reflects baseline biology. As a result, the average body mass index was 23.1 ± 2.0 kg/m^2^, within the normal range.

Parity was generally low (median = 1.0, IQR 0.0–1.0), indicating that most women were either in their first or second pregnancy. Socio-demographic profiling showed that more than 70% were employed, nearly three-quarters had a university or postgraduate degree, and about 80% lived in urban areas, reflecting the setting of a tertiary-care hospital in a large city.

We also excluded participants who fell outside normal ranges for markers of inflammation [18], glucose metabolism [19], micronutrient status [20], iron stores [21], and thyroid function [22] (Table 2). Specifically, CRP ≥ 3 mg/L or IL-6 ≥ 7 pg/mL indicated an inflammatory state capable of promoting telomere erosion via oxidative stress and NF-κB activation [23]. WBC counts < 4.5 or >11 × 10^9^/L were used to rule out infection or marrow dysfunction, both linked to increased cellular turnover [24]. Fasting glucose ≥ 100 mg/dL or HbA1c ≥ 5.7% indicated impaired glycemic control, while ferritin < 15 or >300 ng/mL flagged iron deficiency or overload, both redox-active states that may damage DNA [25]. Vitamin D < 20 ng/mL was excluded because of its association with reduced telomerase activity and systemic inflammation [26]. Finally, TSH outside 0.4–4.0 µIU/mL was considered evidence of thyroid dysfunction, which alters metabolism and reactive oxygen species generation [27]. By excluding these physiological extremes, we established a clinically homogeneous cohort, which allowed for a clearer interpretation of subtle variations in maternal telomere length.

Maternal telomere length, measured by quantitative PCR and expressed as relative T/S ratios, showed considerable variability across the cohort (mean = 0.72 ± 0.60, range 0.47–1.93). This spread is in line with what has been reported in similar populations and reflects the multifactorial influences on telomere dynamics, age, oxidative stress, inflammation, genetic background, and environmental exposures, factors we sought to minimize through strict inclusion criteria, though they cannot be eliminated [23,28,29].

By contrast, the neonatal group was strikingly homogeneous and healthy, consistent with our design. All infants were born at term without congenital anomalies or maternal comorbidities. The mean birthweight was 3284 ± 166 g, with an average length of 52.4 ± 1.7 cm and head circumference of 34.4 ± 1.2 cm. Apgar scores averaged 8.2 ± 0.8 at 1 min and 9.2 ± 0.7 at 5 min, and mean gestational age was 38.9 ± 0.9 weeks.

Correlation analyses revealed robust positive associations between maternal TL and all neonatal parameters assessed. The most pronounced link was with the 1 min Apgar score (r = 0.714, *p* < 0.001), followed by the 5 min Apgar (r = 0.684), length at birth (r = 0.559), and birthweight (r = 0.515). Head circumference (r = 0.468) and gestational age (r = 0.444) also correlated significantly (all *p* < 0.001).

Our findings demonstrate a consistent and biologically plausible relationship between maternal telomere length and key neonatal parameters. The strongest correlation was observed with Apgar scores, particularly at 1 min, which reflects immediate neonatal vitality, cardiorespiratory adaptation, and neurological tone. This suggests that maternal TL, as a marker of biological aging and cumulative oxidative stress, may influence intrauterine conditions that directly affect the newborn’s ability to adapt at birth. Positive associations with birth weight, length, and head circumference further indicate that longer maternal telomeres support adequate fetal growth, possibly through improved placental efficiency, nutrient transfer, and reduced oxidative stress during gestation. The correlation with gestational age, although weaker, reinforces the notion that TL dynamics may modulate the duration of pregnancy by influencing maternal-fetal resilience to stressors that precipitate preterm labor. Taken together, these results highlight maternal TL as an integrative marker linking maternal cellular health to both growth-related and vitality-related neonatal outcomes [8,14].

Partial correlation analyses adjusting for maternal age, BMI, and parity confirmed the robustness of the observed associations. The tertile-based stratification was hypothesis-driven to explore a possible dose–response relationship, and spline regression further suggested a threshold effect, with limited additional benefit in neonatal outcomes above a maternal T/S ratio of ~0.85.

Taken together, these findings suggest that maternal TL, as a molecular marker of cumulative cellular aging and stress, reflects intrauterine conditions with direct impact on fetal growth and early adaptation. Telomere shortening is accelerated by chronic oxidative and inflammatory stress, while longer telomeres appear to signal a more favorable maternal biological profile, with better oxygen and nutrient transfer and improved placental function. The particularly strong correlation with the 1 min Apgar score is clinically important, as this measure captures immediate neonatal vitality, neurological tone, perfusion, and respiration. Given the established links between telomere dynamics, glucocorticoid exposure, and HPA axis function, our results provide a plausible biological bridge between maternal stress biology and neonatal adaptation [30,31,32].

From a mechanistic perspective, maternal oxidative stress, systemic inflammation, and hormonal exposure during pregnancy may represent key biological pathways linking telomere dynamics to neonatal outcomes. Oxidative stress and inflammatory mediators accelerate telomere shortening, potentially compromising placental function, nutrient delivery, and oxygenation. Conversely, longer telomeres may signal a more favorable maternal biological profile, supporting fetal resilience. In addition, maternal glucocorticoid exposure and altered HPA axis regulation have been implicated in fetal programming, with telomere length reflecting cumulative maternal stress biology that can influence neonatal adaptation at birth. Recent studies have also highlighted the role of maternal inflammation in normal pregnancy and its direct impact on fetal growth and development, providing further biological plausibility for our findings [33,34]

In our cohort, all deliveries were performed by cesarean section for maternal indications unrelated to fetal condition, which ensured a uniform mode of delivery and minimized potential variability introduced by intrapartum factors. Therefore, we could not directly compare whether the association between maternal TL and neonatal outcomes differs between cesarean and vaginal deliveries. However, it is reasonable to assume that the observed relationship reflects underlying maternal biology rather than the delivery route, as TL was measured prior to birth and neonatal outcomes assessed immediately thereafter. Future studies including both delivery modes are warranted to clarify whether intrapartum stress or physiological differences between cesarean and vaginal birth might modulate this association. Importantly, our findings were obtained in a clinically healthy maternal population; it is possible that in high-risk pregnancies complicated by obesity, hypertension, diabetes, or systemic inflammation, the relationship between TL and neonatal outcomes may be altered or attenuated, as these conditions are known to accelerate telomere shortening.

Associations between maternal TL and birthweight, length, and head circumference are consistent with previous reports linking shorter maternal or placental telomeres to IUGR, prematurity, and low birthweight. Our findings extend this evidence by analyzing a wider set of neonatal outcomes and by confirming these associations in a Central-European term population, a context where data remain limited [35,36,37].

From a clinical perspective, maternal TL has the potential to serve as a practical biomarker. qPCR assays are inexpensive, require only a peripheral blood sample, and could be integrated alongside routine laboratory tests in late pregnancy. Used in combination with established predictors such as maternal age, BMI, and placental function markers, TL may add value in identifying pregnancies at risk of suboptimal neonatal adaptation even when standard clinical parameters appear normal [14,38].

Compared with established prenatal screening modalities (e.g., first-trimester combined screening with nuchal translucency and serum biochemistry, cfDNA/NIPT, and detailed obstetric ultrasound), maternal TL targets a different clinical construct. Standard tools are designed and validated primarily for aneuploidy and structural anomaly risk stratification, with well-characterized performance metrics and clear clinical pathways. By contrast, maternal TL (measured from peripheral blood) showed consistent associations in our cohort with neonatal vitality (Apgar), growth (birth weight, length, head circumference), and, to a lesser extent, gestational age, positioning it as a potential adjunct marker for perinatal adaptation and growth risk, not a replacement for aneuploidy screening. In this study, TL correlated robustly with multiple neonatal parameters and independently predicted suboptimal 1 min Apgar, supporting construct validity for perinatal risk. However, external validity remains limited by single-center design and the analytical characteristics of relative qPCR (good throughput but lower accuracy vs. gold-standard methods and susceptibility to inter-assay variability). Unlike standard screening tests, clinically actionable cut-offs and reference ranges for TL are not yet established. TL testing is minimally invasive, uses routine venous sampling, and is low-cost and scalable (qPCR). Its envisioned role is add-on risk enrichment late in pregnancy or alongside placental function markers to flag fetuses at risk of suboptimal adaptation despite reassuring routine parameters. It is not suitable as a stand-alone screen for chromosomal or structural anomalies, for which ultrasound/biochemical/cfDNA screening remain appropriate. Like serum biochemistry and cfDNA, TL assessment is non-invasive and poses no procedural risk to mother or fetus. Therefore, adding TL would not increase iatrogenic risk; the main consideration is analytical reliability and clinical interpretation.

Taken together, maternal TL may complement, but not replace, standard prenatal screening by capturing the maternal biological aging/stress axis relevant to fetal growth and immediate neonatal adaptation. Larger, multi-center studies integrating TL with ultrasound, biochemical, and cfDNA parameters should define thresholds, timing, and clinical pathways for implementation

Our results reinforce the concept that maternal biological age, reflected in telomere length, is closely linked to newborn health. Although our observational design cannot establish causality, the strength and consistency of the associations highlight TL as a relevant molecular marker in perinatal research. Importantly, the tertile analysis and logistic regression revealed a threshold effect: mothers with the shortest telomeres had significantly higher odds of delivering newborns with poor early adaptation, while benefits plateaued beyond a T/S ratio of ~0.85. By carefully excluding inflammatory, metabolic, and endocrine confounders, and by combining this with a rich neonatal dataset, our study provides a clearer picture of telomere biology and its clinical relevance [39,40].

If validated in larger and more diverse cohorts, maternal TL could be incorporated into multiparametric screening models to support closer monitoring, targeted preventive measures, and early neonatal care. Such an approach would align with the broader shift towards personalized obstetrics and molecularly informed risk stratification [41].

Our findings highlight maternal TL as a promising candidate biomarker with potential translational value in obstetric care. Given its non-invasive nature, low cost, and technical feasibility through qPCR, TL assessment could be readily integrated into routine prenatal testing alongside existing markers. Rather than replacing established tools such as ultrasound, serum biochemistry, or cfDNA, maternal TL could serve as an adjunct to enrich risk stratification for adverse perinatal outcomes, particularly those related to neonatal growth and adaptation. To move from research to clinical practice, larger multi-center studies are required to establish standardized cut-offs, define optimal timing of assessment, and evaluate cost-effectiveness. Such steps would pave the way for TL-based screening to complement personalized approaches in perinatal medicine.

### Strengths, Limitations and Future Directions

This study benefits from a prospective design, rigorous inclusion and exclusion criteria, and a clinically healthy, term cohort, which collectively reduce major sources of confounding. By trimming participants with obesity, smoking, metabolic disease, overt inflammation, micronutrient deficiency or thyroid dysfunction, we isolated a biologically “clean” sample in which natural variation in TL should be easier to interpret. Telomere assessment followed a standardized qPCR protocol, and the neonatal dataset is unusually rich, capturing weight, length, head circumference, gestational age and dual-time-point Apgar scores for every infant, allowing a nuanced look at multiple aspects of newborn well-being. Finally, the work adds data from a Central-European population that is under-represented in the telomere literature, thereby broadening geographic generalizability.

Our study has several limitations. Being single-center with a moderate sample size (n = 134), its precision and external validity are limited, and replication in larger, multi-ethnic cohorts is needed. The observational, cross-sectional design does not allow causal inference or tracking telomere changes over time. We measured TL only in maternal leukocytes; paternal, placental, and cord-blood telomeres, potential mediators or confounders, were not analyzed. It should also be acknowledged that TL is transmitted from both parents, and placental telomere length reflects the combined maternal and paternal genetic contributions. Our analysis focused solely on maternal TL, which provided insights into maternal biological aging and its intrauterine impact, but did not capture paternal inheritance patterns. Future research should incorporate paternal TL and placental telomere dynamics to clarify the relative influence of both parental lineages on neonatal outcomes. Relative qPCR offered good throughput but less accuracy than gold-standard techniques, and inter-assay variability may have masked small effects. Importantly, a sensitivity analysis excluding outliers in maternal TL and neonatal outcomes confirmed the robustness of our results, strengthening confidence in the reported associations. Residual confounding is also possible, since factors such as psychosocial stress, environmental exposures, or paternal age were not included.

These factors are biologically plausible confounders, as psychosocial stress during pregnancy has been linked to maternal and neonatal telomere dynamics, while environmental exposures such as air pollution and endocrine-disrupting chemicals are established modulators of telomere attrition. Likewise, paternal age contributes to offspring TL at conception and could influence neonatal outcomes independently of maternal biology. Although our strict inclusion criteria reduced many sources of bias, the absence of these variables in our analysis represents a limitation that should be addressed in future multi-factorial studies.

Another limitation comes from the homogeneity of our cohort. We included only healthy women without obesity, smoking, chronic disease, or major metabolic or inflammatory disturbances. This choice reduced confounding but narrows generalizability to higher-risk pregnancies. Likewise, all deliveries were by cesarean section for maternal reasons unrelated to the fetus, to avoid the variability introduced by labor and vaginal birth. While this strengthens internal validity, it further limits broader applicability. Another limitation of our study is that all deliveries were performed by elective cesarean section for maternal indications. While this ensured homogeneity and minimized variability related to intrapartum factors, it may limit generalizability and introduce a degree of selection bias.

Future work should address these gaps. Following mothers and children longitudinally would show whether longer telomeres at birth translate into later developmental or cardiometabolic benefits. Larger, multi-center studies including diverse populations and high-risk pregnancies are also needed. Combining maternal, placental, and cord-blood telomeres with epigenetic clocks or oxidative-stress markers could clarify mechanisms, while interventions such as improved nutrition, vitamin D supplementation, or stress reduction might preserve maternal TL and improve outcomes. Finally, approaches like Mendelian randomization or machine-learning models integrating TL with clinical and omics data could refine causal inference and risk prediction. Together, these steps could move telomere biology from an intriguing correlation toward an actionable tool in perinatal medicine.

## 5. Conclusions

In this prospective cohort, maternal telomere length measured by qPCR showed a clear and consistent relationship with neonatal outcomes. The strongest effect was observed for the 1 min Apgar score, where shorter telomeres independently increased the risk of suboptimal adaptation. Additional positive correlations with birth weight, length, head circumference, and gestational age reinforce the view that maternal telomere dynamics reflect intrauterine conditions with direct consequences for newborn growth and vitality. These results highlight maternal TL as a feasible molecular marker that could complement established predictors in perinatal risk stratification.

## Figures and Tables

**Table 1:** Maternal demographic characteristics.

Variable	Value
Maternal age (years)	28.6 ± 2.2
Body mass index (kg/m^2^)	23.1 ± 2.0
Parity, median (IQR)	1.0 (0.0–1.0)
Education level	
High School	35 (26.1%)
University	75 (56.0%)
Postgraduate	24 (17.9%)
Residence	
Urban	107 (79.9%)
Rural	27 (20.1%)
Employment status	
Employed	96 (71.6%)
Unemployed	23 (17.2%)
Student	15 (11.2%)

**Table 2:** Maternal inflammatory, metabolic, and telomere-related biomarkers.

Parameter	Mean ± SD	95% CI (Lower–Upper)
CRP (mg/L) ^(a)^	1.15 ± 0.56	1.06–1.24
IL-6 (pg/mL) ^(b)^	2.55 ± 1.05	2.37–2.73
WBC (10^9^/L) ^(c)^	6.58 ± 0.89	6.43–6.73
Neutrophils (%)	57.47 ± 5.07	56.61–58.33
Lymphocytes (%)	30.07 ± 4.14	29.37–30.77
Glucose (mg/dL)	89.89 ± 9.58	88.27–91.51
HbA1c (%)	5.26 ± 0.29	5.21–5.31
Ferritin (ng/mL)	71.77 ± 19.63	68.45–75.09
Vitamin D (ng/mL)	35.84 ± 8.28	34.44–37.24
TSH (µIU/mL) ^(d)^	2.02 ± 0.67	1.91–2.13
Telomere Length (relative T/S ratio) ^†^	0.72 ± 0.60	0.626–0.830

^(a)^ C-reactive protein; ^(b)^ Interleukin-6; ^(c)^ White-blood-cell count; ^(d)^ Thyroid-stimulating hormone; ^†^ Measured by quantitative PCR (Cawthon method); values are relative units, not absolute length in base pairs; Relative telomere length was measured using the monochrome qPCR method (Cawthon T/S). Reported values represent relative units (T/S ratio) and do not correspond to absolute telomere length in kilobases or megabases.

**Table 3:** Neonatal characteristics.

Variable	Mean ± SD
Birth Weight (g)	3284 ± 166
Birth Length (cm)	52.4 ± 1.7
Head Circumference (cm)	34.4 ± 1.2
Apgar Score at 1 min	8.2 ± 0.8
Apgar Score at 5 min	9.2 ± 0.7
Gestational Age (weeks)	38.9 ± 0.9

**Table 4:** Neonatal anthropometry and vitality scores stratified by maternal TL tertiles.

Neonatal Parameter	Tertile 1 (TL ≤ 0.60, *n* = 41)	Tertile 2 (0.61–0.85, *n* = 48)	Tertile 3 (TL ≥ 0.86, *n* = 45)	*p*-Value
Apgar—1 min	7.8 ± 0.7	8.3 ± 0.6	8.7 ± 0.5	<0.001
Apgar—5 min	9.0 ± 0.6	9.2 ± 0.6	9.4 ± 0.5	0.004
Birth-weight (g)	3 210 ± 150	3 290 ± 160	3 350 ± 155	0.009
Length (cm)	51.8 ± 1.5	52.4 ± 1.6	52.9 ± 1.4	0.012
Head circumference (cm)	34.0 ± 1.1	34.4 ± 1.2	34.8 ± 1.1	0.018
Gestational age (weeks)	38.7 ± 0.9	38.9 ± 0.8	39.1 ± 0.9	0.060

**Table 5:** Correlation between maternal TL and neonatal parameters.

Neonatal Parameter	Correlation Coefficient (r)	*p*-Value
Birth Weight (g)	0.515	<0.001
Birth Length (cm)	0.559	<0.001
Head Circumference (cm)	0.468	<0.001
Apgar Score at 1 min	0.714	<0.001
Apgar Score at 5 min	0.684	<0.001
Gestational Age (weeks)	0.444	<0.001

**Table 6:** Logistic-regression analysis: predicting a low 1 min Apgar score (<8).

Model	Predictor	Odds Ratio (per +0.25 T/S)	95% CI	*p*-Value
Unadjusted	Maternal TL	0.63	0.48–0.82	0.001
Adjusted ^1^	Maternal TL	0.68	0.51–0.91	0.010

^1^ Adjusted for maternal age (years), body-mass index (kg m^−2^) and parity.

## Data Availability

The data is available from the corresponding author of the study. You may contact the corresponding author for further details and access to the relevant data. Additionally, a copy of the data is also stored in our clinic’s records.
